# *MicroRNA*‐*19a*‐*3p* Decreases with Age in Mice and Humans and Inhibits Osteoblast Senescence

**DOI:** 10.1002/jbm4.10745

**Published:** 2023-04-18

**Authors:** Japneet Kaur, Dominik Saul, Madison L. Doolittle, Joshua N. Farr, Sundeep Khosla, David G. Monroe

**Affiliations:** ^1^ Division of Endocrinology, Department of Medicine Mayo Clinic College of Medicine Rochester MN USA; ^2^ Robert and Arlene Kogod Center on Aging Mayo Clinic Rochester MN USA

**Keywords:** AGING, CELLS OF BONE, OSTEOBLASTS

## Abstract

Aging is a major risk factor for most chronic diseases, including osteoporosis, and is characterized by an accumulation of senescent cells in various tissues. MicroRNAs (miRNAs) are critical regulators of bone aging and cellular senescence. Here, we report that *miR*‐*19a*‐*3p* decreases with age in bone samples from mice as well as in posterior iliac crest bone biopsies of younger versus older healthy women. *miR*‐*19a*‐*3p* also decreased in mouse bone marrow stromal cells following induction of senescence using etoposide, H_2_O_2_, or serial passaging. To explore the transcriptomic effects of *miR*‐*19a*‐*3p*, we performed RNA sequencing of mouse calvarial osteoblasts transfected with control or *miR*‐*19a*‐*3p* mimics and found that *miR*‐*19a*‐*3p* overexpression significantly altered the expression of various senescence, senescence‐associated secretory phenotype‐related, and proliferation genes. Specifically, *miR*‐*19a*‐*3p* overexpression in nonsenescent osteoblasts significantly suppressed *p16* ^*Ink4a*^ and *p21* ^*Cip1*^ gene expression and increased their proliferative capacity. Finally, we established a novel senotherapeutic role for this miRNA by treating *miR*‐*19a*‐*3p* expressing cells with H_2_O_2_ to induce senescence. Interestingly, these cells exhibited lower *p16* ^*Ink4a*^ and *p21* ^*Cip1*^ expression, increased proliferation‐related gene expression, and reduced SA‐β‐Gal+ cells. Our results thus establish that *miR*‐*19a*‐*3p* is a senescence‐associated miRNA that decreases with age in mouse and human bones and is a potential senotherapeutic target for age‐related bone loss. © 2023 The Authors. *JBMR Plus* published by Wiley Periodicals LLC on behalf of American Society for Bone and Mineral Research.

## Graphical abstract

Physiological aging or DNA‐damaging agents increase expression of *p16* ^*Ink4a*^ and *p21* ^*Cip1*^ expression and SA‐β‐Gal cell activity in osteoblasts, while simultaneously decreasing *miR*‐*19a*‐*3p* expression. Restoring *miR*‐*19a*‐*3p* expression using a *miR*‐*19a*‐*3p* mimic attenuates both *p16* ^*Ink4a*^ and *p21* ^*Cip1*^ expression, as well as SA‐β‐Gal cell activity, in senescent osteoblasts.

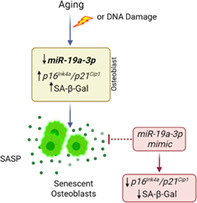

## Introduction

Aging is a predominant risk factor for most chronic diseases such as osteoporosis, sarcopenia, diabetes, Alzheimer's, cardiovascular disorders, and cancer and results in progressive multiorgan deterioration and tissue dysfunction that ultimately shortens both lifespan and healthspan.^(^ ^1^, ^2^, ^3^, ^4^, ^5^, ^6^ ^)^ The Geroscience Hypothesis states that manipulation of the fundamental mechanisms of aging, rather than treating each disorder symptomatically, could delay or alleviate the effects of these conditions.^(^ ^7^ ^)^ Cellular senescence is one such aging mechanism that can be targeted using drugs called senotherapeutics to prevent or treat multiple age‐associated diseases and improve healthspan, to potentially extend years of life free of disease.^(^ ^8^, ^9^, ^10^, ^11^ ^)^

Cellular senescence is a cell fate program that causes permanent cell cycle arrest in response to a stressor, such as DNA damage, oncogenic insults, or increased reactive oxygen species. Other characteristics of cellular senescence include activation of tumor suppressor pathways (primarily the *p53/p21* ^*Cip1*^ and *p16* ^*Ink4a*^ */Rb* pathways), changes in chromatin organization, resistance to apoptosis, and excessive production of a proinflammatory secretome (i.e., the senescence‐associated secretory phenotype [SASP]) that spreads senescence to neighboring healthy cells and tissues. Senescent cells are further characterized by their flattened morphology and increased senescence‐associated β‐galactosidase (SA‐β‐Gal) activity.^(^ ^8^, ^12^, ^13^, ^14^, ^15^ ^)^ Aging leads to the accumulation of senescent cells in various tissues that contribute to increased severity of chronic diseases, including osteoporosis, that can be attenuated following selective removal of these dysfunctional cells.^(^ ^16^, ^17^, ^18^ ^)^ This has led to mounting interest in identifying novel agents that can selectively kill senescent cells (i.e., senolytics) or suppress their SASP (i.e., senomorphics) and assessing their efficacy in improving healthspan.^(^ ^19^ ^)^

Recently, miRNAs have emerged as important regulators of bone remodeling, aging, and cellular senescence and are known to be mechanistically involved in regulating a wide range of biological functions such as cell proliferation, growth, apoptosis, regeneration, and metabolism.^(^ ^20^, ^21^, ^22^, ^23^, ^24^ ^)^ miRNAs are a class of small evolutionarily conserved noncoding RNAs (~22 nucleotides) that recognize and pair to the 3′‐untranslated regions (3'‐UTR) of target mRNAs, resulting in degradation or translational suppression.^(^ ^21^, ^25^ ^)^ They possess the ability to stably circulate throughout the body, making them potent signaling molecules, disease biomarkers, and essential prognostic and therapeutic tools in various diseases.^(^ ^26^, ^27^, ^28^, ^29^, ^30^ ^)^ Particularly, the ability of a single miRNA to simultaneously suppress multiple target mRNAs, thereby effectively regulating numerous genes simultaneously within key pathways based on the physiological or disease state of the body, makes them attractive, although incompletely explored, candidates for miRNA‐based therapeutics in age‐related conditions.^(^ ^31^, ^32^, ^33^, ^34^ ^)^

With the mounting interest in the role of miRNAs in bone aging and cellular senescence, we aimed to identify miRNAs whose expression changed with both aging and senescence and determine whether they possessed senotherapeutic effects in vitro To this end, we identified *miR*‐*19a*‐*3p*, a miRNA previously shown to have effects in mitigating age‐related bone loss in mice^(^ ^35^ ^)^ and in regulating mesenchymal stem cell differentiation,^(^ ^36^ ^)^ as having potential antisenescence effects in cultured mouse bone cells.

## Materials and Methods

### Bone tissue samples (mouse and human)

All mouse studies were conducted in accordance with National Institutes of Health (NIH) guidelines and approved by the Institutional Animal Care and Use Committee at the Mayo Clinic. All mice in the C57BL/6N background were purchased from Charles River Laboratories (Wilmington, MA, USA). The vertebrae were isolated from both 6‐ and 24‐month‐old male mice (*n* = 10/group). The bone tissue was cleaned of associated muscle and connective tissues, minced into ~1‐mm pieces, and sequentially digested twice for 30 minutes in endotoxin‐free collagenase (Liberase; Roche Diagnostics GmbH, Mannheim, Germany) to obtain osteocyte‐enriched bone samples. These were then homogenized in QIAzol reagent (Qiagen, Valencia, CA) for total RNA isolation and used for miRNA sequencing.^(^ ^16^ ^)^

The human bone biopsy samples used in this study were isolated as previously described.^(^ ^16^, ^17^, ^37^ ^)^ They were small needle bone biopsies isolated from the posterior iliac crest procured from 10 young premenopausal women (mean age ± SD, 27 ± 3 years; range 23 to 30 years) and 10 old postmenopausal women (78 ± 5 years; range 72 to 87 years). Postmenopausal status was established by the absence of menses for >1 year and serum follicle‐stimulating hormone levels >20 IU/L. Extensive exclusion criteria for these patients and the study protocol are as previously described.^(^ ^16^ ^)^ All human studies were approved by the Mayo Clinic Institutional Review Board.

### Cell culture and cell treatments

Mouse bone marrow stromal cells (BMSCs) were isolated from 3‐month‐old C57BL/6N mice after removing the epiphyseal growth plates from the tibiae and femora and flushed with Dulbecco's Modified Eagle Medium (Thermo Fisher Scientific, Waltham, MA, USA) supplemented with 1× antibiotic/antimycotic (Thermo Fisher Scientific, Waltham, MA, USA), 1× Glutamax, and 15% (v/v) fetal bovine serum (GE Healthcare Life Sciences HyClone Laboratories, Logan, UT, USA). Half media change was performed on day 3, and the cells were plated for the experiment on day 7 at a cell density of 1 × 10^4^ cells/cm^2^ in 12‐well tissue culture plates and allowed to grow to 70% to 80% confluence. To induce senescence, the cells were then treated with (i) 20 uM etoposide (MilliporeSigma, St. Louis, MO, USA) or vehicle (0.1% dimethyl sulfoxide) for 48 hours followed by maintenance in growth media for 6 days and (ii) 500 uM H_2_O_2_ for 20 minutes, followed by maintenance in growth media for 4 days, and then another treatment with 500 uM H_2_O_2_ for 20 minutes; (iii) the cells were subcultured until they stopped dividing and became permanently growth arrested or senescent (about eight passages). Cells were lysed in QIAzol reagent (Qiagen) for RNA isolation or fixed with 4% paraformaldehyde for SA‐β‐Gal assay.

Mouse calvarial osteoblasts (CalOBs) were harvested as previously described.^(^ ^38^ ^)^ Briefly, 1‐ to 3‐day‐old C57BL/6N pups were used to isolate cells from the mouse calvarium by performing serial digests in collagenase type 2 (4 mg/mL) and bovine serum albumin (4 mg/mL) for 10 minutes at 37°C. The third fraction was collected and cultured in alpha‐minimal essential growth medium supplemented with 1× antibiotic/antimycotic, 1× Glutamax, and 10% (v/v) fetal bovine serum. Senescence induction using etoposide or H_2_O_2_ was performed as for the mouse BMSCs.

### miRNA sequencing

miRNA‐sequence analysis was performed on the osteocyte‐enriched bone samples obtained from collagenase‐digested vertebrae of young (*n* = 10, 6 months) and old (*n* = 10, 24 months) C57BL/6N male mice using previously described methods.^(^ ^16^ ^)^ MicroRNAs with raw read counts less than 4 in both groups were classified as unreliable and deleted. Analysis was performed using the edgeR package in R, and normalized expression was used to determine the log fold‐change (FC). A subset of miRNAs fitting the following criteria were selected for further analyses: *p*_adjus_ < 0.05, log FC > [1], and predicted to regulate the cellular senescence pathway using Kyoto Encyclopedia of Genes and Genomes (KEGG) pathway analyses (*p* < 0.05). This miRNA‐sequencing data are accessible through the Gene Expression Omnibus (GEO) accession no. GSE226402.

### miRNA mimic transfection and mRNA sequencing of CalOBs

Primary mouse CalOBs were used and cultured as described earlier. The transfection efficiency of these cells was first examined by transfecting 10 nM of a negative control or BLOCK‐iT™ Alexa Fluor™ Red Fluorescent Control (Invitrogen, Waltham, MA, USA) siRNA using RNAiMax reagent (Invitrogen), and we confirmed nearly 100% transfection efficiency at 48 hours following transfection using a fluorescence microscope (Evos M5000, Invitrogen). Note that siRNAs and miRNAs are the same length (~21 nucleotides), so this siRNA control was valid in assessing the transfection efficiency of miRNAs. For the RNA‐sequencing experiment, 1 × 10^6^ CalOBs were identically transfected with 10 nM of either negative control or *miR*‐*19a*‐*3p* miRNA mimic (*n* = 3) and harvested in QIAzol (Qiagen) 48 hours later. For experiments described in Fig. 4, cells were harvested at the additional time points of 24 and 72 hours following transfection. For the experiments described in Fig. 5, following transfection with negative control and *miR*‐*19a*‐*3p* mimics, the cells were either maintained in growth medium or exposed to H_2_O_2_ to induce cellular senescence.

RNA sequencing was performed on a HiSeq4000 (Illumina, Sand Diego, CA, USA) using TruSeq SBS sequencing software (version 3) and SCS data collection software (version 1.4.8). Fastq files were mapped to the murine reference genome mm10, and analysis was performed as previously described.^(^ ^17^, ^39^ ^)^ Briefly, the R package DeSeq2^(^ ^40^ ^)^ was used to identify the differentially expressed genes between the negative control and *miR*‐*19a*‐*3p*‐transfected samples. Comparison between groups was based on negative binomial distribution, and the multiple testing problem was corrected using the Benjamini–Hochberg procedure. Specifically, the Gene Set Enrichment Analysis (GSEA) analysis was based on the FC of *miR*‐*19a*‐*3p*‐transfected cells compared to control cells and KEGG pathway analysis was performed on the genes that were significantly differentially up‐ and downregulated by *miR*‐*19a*‐*3p* expression. These mRNA‐sequencing data are accessible through the GEO accession no. GSE226537.

### SA‐β‐Gal assay

Cellular SA‐β‐Gal activity was assayed as previously described.^(^ ^41^ ^)^ In brief, following fixation, the BMSCs were washed three times with 1× PBS before being incubated in SA‐β‐Gal activity solution (pH 6.0) at 37°C for 16 to 18 hours. The enzymatic reaction was stopped by washing cells or tissues three to five times with ice‐cold 1× PBS and visualized using light microscopy.

### RT‐qPCR

Total RNA (125 ng) was used to generate cDNA using the High‐Capacity cDNA Reverse Transcription Kit (Applied Biosystems, Carlsbad, CA, USA) according to the manufacturer's instructions. qPCR analysis was performed using the ABI Prism 7900HT Real‐Time System instrument (Applied Biosystems) with SYBR Green reagent (Qiagen), as previously described.^(^ ^17^, ^42^ ^)^ Data normalization was performed using three reference genes (*Actb*, *Hprt*, *Tuba1a*), and threshold calculations were based on their stability, as previously described.^(^ ^43^ ^)^ The oligonucleotide sequences for the genes measured in this study were designed using the Primer Express program (Applied Biosystems) and can be found in Table 1.

**Table 1:** RT‐qPCR Primer Sequences Used in This Study

Gene symbol	Forward primer (5′ to 3′)	Reverse primer (5′ to 3′)
*Cdkn1a* (*p16* ^*Cip1*^)	GAACATCTCAGGGCCGAAAA	TGCGCTTGGAGTGATAGAAATC
*Cdkn2a* (*p16* ^*Ink4a*^)	GAACTCTTTCGGTCGTACCC	AGTTCGAATCTGCACCGTAGT
*Mki67* (*Ki67*)	AGACTGCCTCCCAGGAGACA	GGCCCCGAGATGTAGATTTCT
*Actb*	AATCGTGCGTGACATCAAAGAG	GCCATCTCCTGCTCGAAGTC
*Hprt*	CGTGATTAGCGATGATGAACCA	TCCAAATCCTCGGCATAATGA
*Tuba1a*	GGTTCCCAAAGATGTCAATGCT	CAAACTGGATGGTACGCTTGGT

For measuring miRNA expression, total RNA (100 ng), including the miRNA fraction, was reverse‐transcribed using the miRCURY LNA RT Kit (Qiagen) as per the manufacturer's protocol. The individual miRNA assays used in this study were purchased from Qiagen and used with the miRCURY LNA miRNA PCR Starter Kit (Qiagen) according to the manufacturer's instructions. Data normalization was performed using the *Let*‐*7f*‐*5p*.^(^ ^44^ ^)^

### Cell proliferation assay

Primary mouse CalOBs were seeded in growth medium into 96‐well plates at a density of 2 × 10^4^ cells/cm^2^ (*n* = 6) and allowed to proliferate for 24 hours. The cells were then transfected with *miR*‐*19a*‐*3p* and negative control mimic (NC mimic) miRNA mimics for 48 hours. Twenty‐five (25) μL of conditioned medium was assayed using the CellTiter‐Glo® Luminescent Cell Viability Assay (Promega, Madison, WI, USA) according to the manufacturer's instructions. The plate was read on a GloMax® luminometer (Promega) and data expressed as luciferase activity relative to NC mimic. Cells were visualized using crystal violet staining, fixed in 4% formaldehyde in 1× PBS for 20 minutes, and, upon washing with 1× PBS, stained with 1% (w/v) crystal violet in 20% ethanol for 20 minutes. Excess dye was removed and upon drying, and images were acquired.

### Data analysis

All values are expressed as mean ± SEM. Mean values were compared between two groups using an independent samples *t* test. Differences were considered significant at *p* < 0.05 (two‐tailed test). miRNA and mRNA expression levels of each sample were determined by CT values measured in triplicate. The FCs were determined using the relative quantification method (2^−ΔΔCt^) with the selected endogenous control. Analyses were performed using the Statistical Package for the Social Sciences for Windows (version 25.0; SPSS, Chicago, IL, USA); figures were created using GraphPad Prism (version 8.3.1; GraphPad Software, San Diego, CA, USA).

## Results

### *miR* ‐*19a*‐*3p* expression decreases with aging in mice and humans

**Fig. 1:**
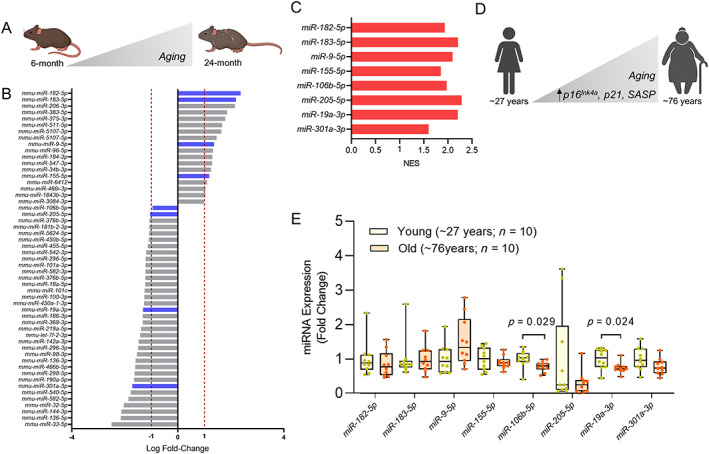
Differentially expressed age‐related miRNAs. (*A*) Schematic diagram of design for miRNA sequence data in mice (designed using Biorender.com). (*B*) miRNA sequence data showing miRNAs with log fold‐change > [1] that were differentially expressed in bone samples from vertebrae of young (6‐month) and old (24‐month) male mice (*n* = 10/group). The bars colored in blue highlight the miRNAs that were predicted to regulate the cellular senescence pathway using in silico analyses. (*C*) Normalized enrichment score (NES) for miRNAs differentially expressed with aging and predicted to target cellular senescence pathway (blue bars in *B*). (*D*) Schematic diagram of design for human bone biopsies from posterior iliac crest (designed using Biorender.com). (*E*) RT‐qPCR expression of miRNAs predicted to regulate cellular senescence was assessed in needle bone biopsies from posterior iliac crest of young (mean ± SD; 27 ± 3 years) and old (mean ± SD; 78 ± 6 years) healthy female volunteers (*n* = 10/group). Values of *p* are shown numerically with *p* < 0.05 (independent samples *t* test).

To identify miRNAs that might be involved in aging and cellular senescence, we first examined a miRNA sequence dataset comparing vertebral bone of young (6‐month‐old) and old (24‐month‐old) mice (Fig. 1*A*).^(^ ^44^ ^)^ We selected the miRNAs with a log FC > [1] (Fig. 1*B*) that were significantly predicted to target the cellular senescence pathway (Fig. 1 *B*, blue bars) using a normalized enrichment score (Fig. 1*C*). *miRs*‐*182*‐*5p*, *−183*‐*5p*, *−9*‐*5p*, and *−155*‐*5p* all increased significantly with aging, whereas *miRs*‐*106b*‐*5p*, *−205*‐*5p*, *−19a*‐*3p*, *and* ‐*301a*‐*3p* all decreased with aging in mice (Fig. 1*B*).

Next, we assessed wether these miRNAs were also altered with aging in human bone biopsies. Their change in expression was measured using RT‐qPCR in needle bone biopsies from the posterior iliac crest of younger (mean ± SD = 27 ± 3 years) and older (mean ± SD = 78 ± 6 years) healthy women who had previously been shown to have significantly higher expression of senescence‐related genes, *p16* ^*Ink4a*^ and *p21* ^*Cip1*^, and certain SASP factors (Fig. 1*D*).^(^ ^16^ ^)^ Of the candidate miRNAs identified in vertebral bone of mice, we found that *miRs*‐*19a*‐*3p* and *miRs*‐*106b*‐*5p* also decreased significantly with aging in human bone (Fig. 1*E*), in a manner and direction similar to those in the mouse bone dataset.

### *miR* ‐*19a*‐*3p* expression decreases with senescence in BMSCs

**Fig. 2:**
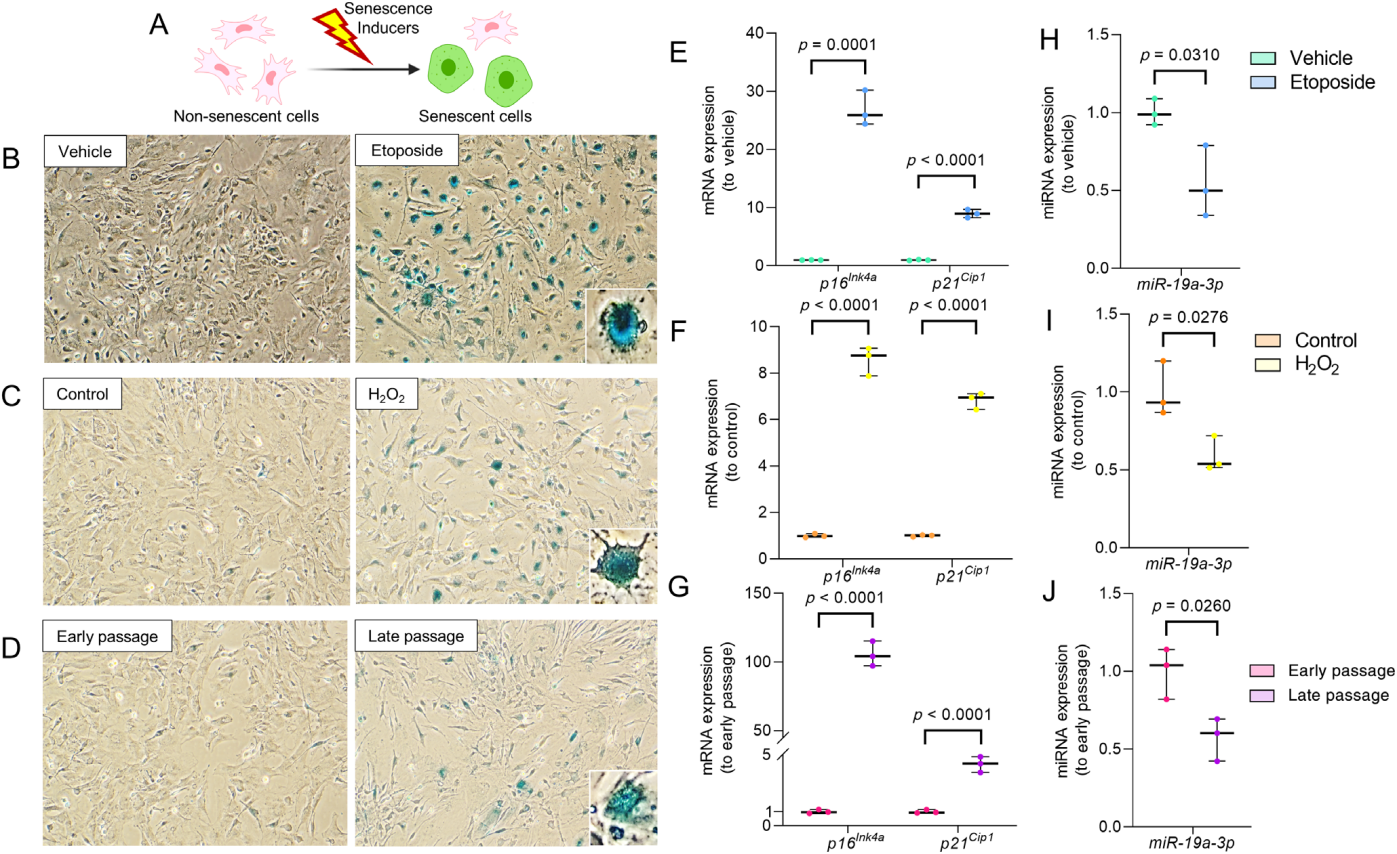
Change in expression of *miR*‐*19a*‐*3p* with senescence. (*A*) Schematic diagram of design showing induction of senescence following DNA damage (designed using Biorender.com). Representative images of SA‐β‐Gal‐stained BMSCs treated with (*B*) vehicle (DMSO) and etoposide (20 uM), (*C*) control (untreated) and H_2_O_2_, (*D*) early and late passage (magnification ×10; *n* = 3/group). RT‐qPCR analysis of (*E*–*G*) *p16* ^*Ink4a*^ and *p21* ^*Cip1*^ and (*H*–*J*) *miR*‐*19a*‐*3p* in nonsenescent and senescent BMSCs. Gene expression was denoted as fold‐change relative to vehicle or control (*n* = 3/group). Values of *p* are shown numerically with *p* < 0.05 (independent samples *t* test). BMSC = bone marrow stromal cell; DMSO = dimethyl sulfoxide.

As *miR*‐*19a*‐*3p* and *miR*‐*106b*‐*5p* both decreased significantly with aging in mice and human bone, we next examined whether their expression was altered with cellular senescence in mouse BMSCs. We utilized several different inducers of senescence, such as etoposide, H_2_O_2_, and serial passaging, to induce senescence in BMSCs (Fig. 2*A*). Induction of senescence with each separate inducer was confirmed by increased SA‐β‐Gal activity (Fig. 2*B*–*D*) and upregulation of *p16* ^*Ink4a*^ and *p21* ^*Cip1*^ gene expression (Fig. 2*E*–*G*). Importantly, *miR*‐*19a*‐*3p* expression decreased with senescence with each of these three inducers (Fig. 2*H*–*J*). However, there was no change in the expression of *miR*‐*106b*‐*5p* with the induction of cellular senescence (Fig. S1A–C). Because *miR*‐*19a*‐*3p* had robust alterations in response to both aging in vivo and senescence in vitro, we focused our further experiments and analyses on *miR*‐*19a*‐*3p*.

### Effects of *miR* ‐*19a*‐*3p* on cellular senescence gene expression

We followed an unbiased approach to explore the mRNA targets of *miR*‐*19a*‐*3p* in bone cells and performed RNA sequencing (RNAseq) of CalOBs transfected with negative control or *miR*‐*19a*‐*3p* miRNA mimics for 48 hours. CalOBs were chosen for the RNAseq analysis since they have high transfection efficiency, can be transfected without affecting cell number or altering cellular activity, and are less heterogeneous than BMSCs. Moreover, we found that CalOBs are a robust model for inducing senescence using either etoposide or H_2_O_2_, resulting in enhanced SA‐β‐Gal activity (Fig. S2*A*, *B*) and increased *p16* ^*Ink4a*^ and *p21* ^*Cip1*^ gene expression (Fig. S2*C*, *D*), similar to what we found in BMSCs (Fig. 2).

**Fig. 3:**
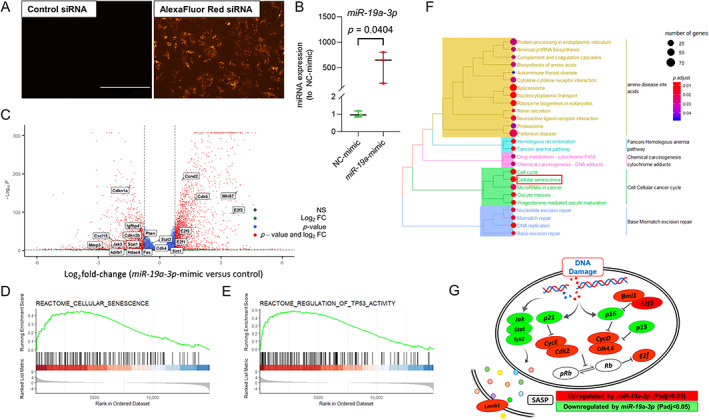
mRNA targets of *miR*‐*19a*‐*3p*. (*A*) To assess transfection efficiency of miRNA‐sized nucleic acids, CalOBs were transfected with a nonlabeled control or an Alexa Fluor Red‐labeled fluorescent siRNA (same size as miRNAs) and visualized using fluorescence microscopy (555 nm). (*B*) CalOBs were transfected with negative control or *miR*‐*19a*‐*3p* miRNA mimics and expression measured by RT‐qPCR at 48 hours after transfection. (*C*) Volcano plot of RNAseq data highlighting selected genes that change with *miR*‐*19a*‐*3p* overexpression compared to control (*p*_adj_ < 0.05; log_2_ FC > [1]). GSEA was performed on RNAseq data and showed enrichment for genes associated with (*D*) cellular senescence and (*E*) TP53 activity. (*F*) KEGG pathway analysis of significantly expressed genes shows regulation of cellular senescence pathway by *miR*‐*19a*‐*3p*. (*G*) Schematic highlighting some key players in cellular senescence pathway whose gene expression was significantly altered by *miR*‐*19a*‐*3p*. Chronic DNA damage ultimately results in increased *p16* ^*Ink4a*^ *and p21* ^*Cip1*^ activity and subsequently decreased cell proliferation. This is accompanied by production of proinflammatory SASP driven by Janus kinase (JAK) signal transducer and activator of transcription (STAT) pathway that spreads senescence to neighboring healthy cells. The figure depicts the effects of *miR*‐*19a*‐*3p* overexpression on each of these genes using RNAseq data. RT‐qPCR was used to assess *p16* ^*Ink4a*^ gene expression. Values of *p* are shown numerically with *p* < 0.05 (independent samples *t* test). CalOBs = calvarial osteoblasts; FC = fold‐change; GSEA = Gene Set Enrichment Analysis; SASP = senescence‐associated secretory phenotype.

To assess the transfection efficiency of CalOBs, we transfected a negative control or fluorescently labeled siRNA (similar in length to miRNAs) into CalOBs and determined that nearly all the cells were transfected at 48 hours after transfection (Fig. 3*A*). Using identical methods, we transfected either a negative control or *miR*‐*19a*‐*3p* miRNA mimics and confirmed overexpression using RT‐qPCR at 48 hours (Fig. 3*B*). RNAseq analysis was then performed on these samples (see “Materials and Methods” for details). The volcano plot constructed from this RNAseq dataset highlights selected genes associated with senescence, SASP, and cell proliferation that change significantly (*p*_adjusted_ < 0.05) with *miR*‐*19a*‐*3p* overexpression (Fig. 3*C*).

We then utilized GSEA to evaluate the overall trends for differences in gene expression between the control and *miR*‐*19a*‐*3p‐*mimic‐transfected cells (Table S1). We noted that *miR*‐*19a*‐*3p* significantly regulated the expression of genes involved in cellular senescence (Fig. 3*D*; Fig. S3*A*) and regulation of *p53* activity (Fig. 3*E*; Fig. S3*B*). GSEA results were consistent with KEGG pathway analysis showing that *miR*‐*19a*‐*3p* significantly regulated the cellular senescence pathway (Fig. 3*F*; Table S2). Next, we created a list of all predicted targets of *miR*‐*19a*‐*3p* using TargetScanMouse 8.0^(^ ^45^, ^46^ ^)^ and present the data for those targets from our RNAseq data (Tables S1 and S3). We further narrowed the targets with those associated with senescence and SASP by curating a list of senescence‐ and SASP‐related genes based on the existing literature^(^ ^47^, ^48^ ^)^ and matching that to the direct targets of *miR*‐*19a*‐*3p* (Tables S2 and S3). Overall, the RNAseq data demonstrated that increased expression of *miR*‐*19a*‐*3p* downregulated some of the key senescence‐ and SASP‐related genes and upregulated genes related to cell proliferation, indicating its potential role in the inhibition of cellular senescence (Fig. 3*G*; Fig. S3*C*).

**Fig. 4:**
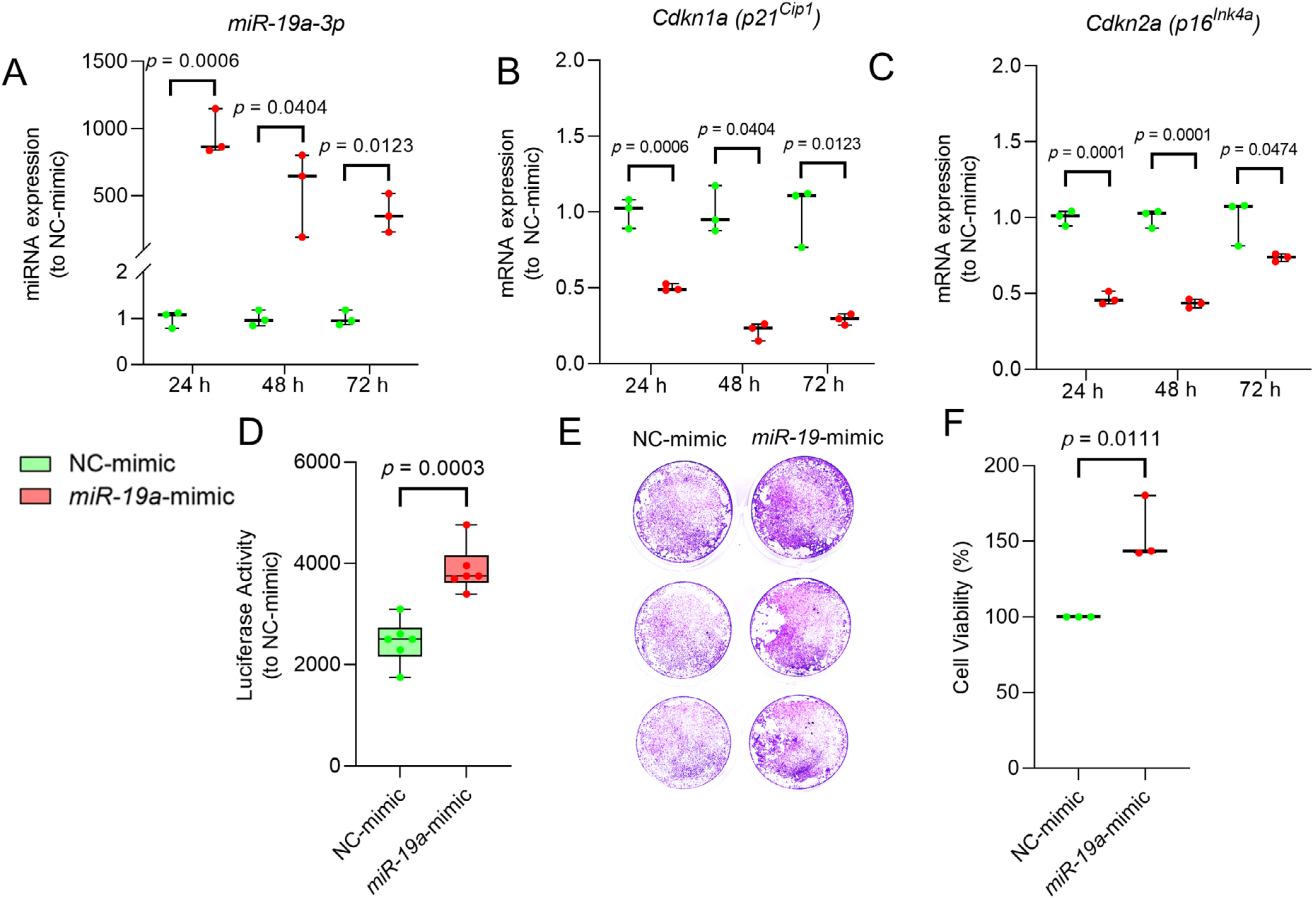
Effects of *miR*‐*19a*‐*3p* on *p16* ^*Ink4a*^ *and p21* ^*Cip1*^ gene expression and proliferation. RT‐qPCR analysis of (*A*) *miR*‐*19a*‐*3p*, (*B*) *p21* ^*Cip1*^, and (*C*) *p16* ^*Ink4a*^ in control (NC mimic) and *miR*‐*19a*‐*3p* mimic transfected cells. Proliferative capacity of CalOBs transfected with control or *miR*‐*19a*‐*3p* miRNA mimics (*n* = 3/group) as shown by (*D*) cell proliferation assay, (*E*) crystal violet stain, and (*F*) percentage cell viability. Values of *p* are shown numerically with *p* < 0.05 (independent samples *t* test). CalOBs = calvarial osteoblasts; NC = negative control.

Next, we measured the overexpression of *miR*‐*19a*‐*3p* (Fig. 4*A*) and change in mRNA levels of key senescence genes, *p21* ^*Cip1*^ (Fig. 4*B*) and *p16* ^*Ink4a*^ (Fig. 4*C*), using RT‐qPCR in CalOBs. We found that both *p16* ^*Ink4a*^ and *p21* ^*Cip1*^ were downregulated using RT‐qPCR. Furthermore, *miR*‐*19a*‐*3p*‐mimic‐transfected cells had higher proliferative capacity (Fig. 4*D*), a stronger crystal violet stain (Fig. 4*E*), and a higher percentage of viable cells (Fig. 4*F*) compared to control cells.

### *miR* ‐*19a*‐*3p* attenuates the effects of cellular senescence in osteoblasts

**Fig. 5:**
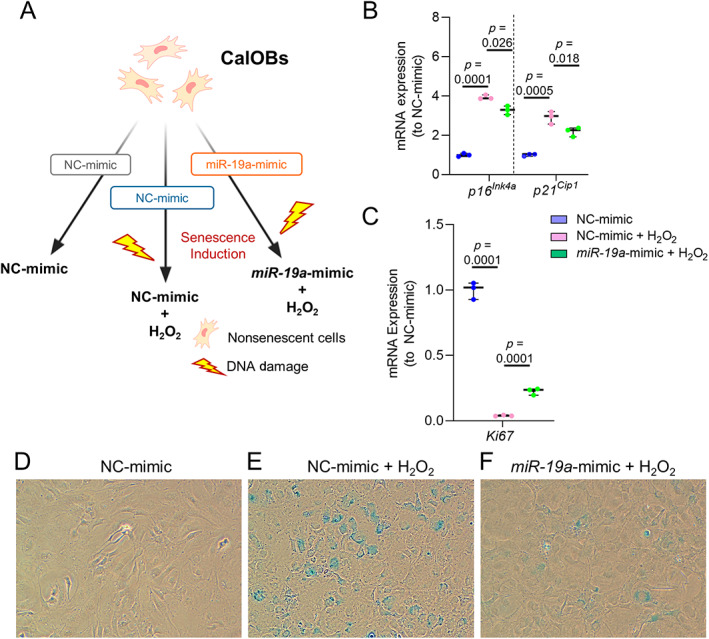
Senotherapeutic effects of *miR*‐*19a*‐*3p*. (*A*) Calvarial osteoblasts were transfected with control (NC mimic) and *miR*‐*19a*‐*3p* mimic for 48 hours, followed by senescence induction using H_2_O_2_ in groups indicated in schematic diagram (designed using Biorender.com). RT‐qPCR analysis of *p16* ^*Ink4a*^ and *p21* ^*Cip1*^ in (*B*) NC mimic, NC mimic + H_2_O_2_, and *miR*‐*19a* mimic + H_2_O_2_ transfected and treated cells (*n* = 3/group). RT‐qPCR analysis of *Ki67* in (*C*) NC mimic, NC mimic + H_2_O_2_, and *miR*‐*19a* mimic transfected and treated cells (*n* = 3/group). Representative images of SA‐β‐Gal‐stained cells, (*D*) NC mimic, (*E*) NC mimic + H_2_O_2_, and (*F*) *miR*‐*19a* mimic + H_2_O_2_ (*n* = 3/group; magnification ×10). Values of *p* are shown numerically with *p* < 0.05 (independent samples *t* test). NC = negative control.

Finally, we tested the senotherapeutic potential of *miR*‐*19a*‐*3p* by exposing control and *miR*‐*19a*‐*3p*‐mimic‐transfected CalOBs to H_2_O_2_ to induce senescence (Fig. 5*A*). We found that cells transfected with NC mimic and exposed to H_2_O_2_ treatment (NC mimic + H_2_O_2_) became senescent and had increased *p16* ^*Ink4a*^ and *p21* ^*Cip1*^ gene expression compared to non‐H_2_O_2_‐treated cells (NC mimic), while prior transfection of *miR*‐*19a*‐*3p* (*miR*‐*19a* mimic + H_2_O_2_) reduced expression of these senescence markers compared to NC mimic + H_2_O_2_ (Fig. 5*B*). *Ki67* expression, which is a marker of cellular proliferation, significantly decreased in NC mimic cells treated with H_2_O_2_, which was reversed with prior transfection of *miR*‐*19a*‐*3p* (*miR*‐*19a* mimic + H_2_O_2_) (Fig. 5*C*). Although the reversal of gene expression patterns elicited by prior transfection of *miR*‐*19a*‐*3p* did not return to the level of NC mimic (no H_2_O_2_) alone, the results still demonstrated that *miR*‐*19a*‐*3p* had modest, but statistically significant, effects on senoprotection in CalOBs. Similarly, *miR*‐*19a*‐*3p*‐mimic‐transfected cells resulted in fewer SA‐β‐Gal‐positive cells than NC mimic cells treated with H_2_O_2_ (Fig. 5*D*–*F*). These results demonstrate that increased *miR*‐*19a*‐*3p* levels can protect osteoblasts from the deleterious effects of cellular senescence.

## Discussion

The goal of this study was to identify miRNAs associated with aging and cellular senescence that might inhibit or delay senescence and thereby attenuate the aging process in bone. We used a systematic approach and utilized miRNA sequence data to select a subset of miRNAs whose expression changed with age in bone tissue from young and old mice and were predicted to regulate the cellular senescence pathway. Of the miRNAs tested, we found that *miR*‐*19a*‐*3p* decreased with age in the bone of both mice and humans and following induction of senescence in BMSCs. Moreover, cells overexpressing *miR*‐*19a*‐*3p* had decreased senescence effector (i.e., *p16* ^*Ink4a*^ and *p21* ^*Cip1*^) gene expression and exhibited higher proliferative capacity. We established its senotherapeutic role by treating *miR*‐*19a*‐*3p*‐overexpressing cells with H_2_O_2_ to induce senescence. Cells expressing *miR*‐*19a*‐*3p* had lower *p16* ^*Ink4a*^ and *p21* ^*Cip1*^ mRNA levels, an increase in *Ki67* gene expression, and fewer SA‐β‐Gal+ cells. Our results thus establish that *miR*‐*19a*‐*3p* is an aging‐ and senescence‐associated miRNA in vivo and in vitro and a potential senotherapeutic.

*miR*‐*19a*‐*3p* is a part of the previously identified *miR*‐*17*‐*92* cluster and resides on chromosome 14 in mice and chromosome 13 in humans. This cluster comprises six miRNAs (*miR*‐*17*, *miR*‐*18a*, *miR*‐*19a*, *miR*‐*20a*, *miR*‐*19b*‐*1*, *and miR*‐*92a*‐*1*) and is highly conserved among vertebrates.^(^ ^49^, ^50^, ^51^ ^)^ Interestingly, many miRNAs in this cluster are known to be downregulated with aging, and increased expression of *miR*‐*17*, *miR*‐*19b*, *miR*‐*20a*, *and miR*‐*106a* has been associated with the downregulation of *p21* ^*Cip1*^ mRNA levels; however, these analyses were performed in nonbone systems.^(^ ^52^, ^53^ ^)^ Chen et al.^(^ ^35^ ^)^ showed that *miR*‐*19a*‐*3p* decreased with age in cultured BMSCs from both humans and mice. We demonstrate here that this decrease in cultured cells is also present in vivo using bone samples from mice and humans. Further, this decrease correlated with increased levels of the cyclin D kinase inhibitors, *p16* ^*Ink4a*^ and *p21* ^*Cip1*^, and certain SASP factors. In addition, *miR*‐*19a*‐*3p* expression was also decreased in BMSCs following induction of cellular senescence with a variety of stressors, such as etoposide, passaging, and H_2_O_2_, compared to control cells. In contrast to our findings and those of Chen et al., Kangas et al. reported that *miR*‐*19a*‐*3p* was upregulated with age in the adipose tissue of postmenopausal women compared to their younger premenopausal counterparts; however, no change in expression was apparent in its circulating plasma levels.^(^ ^35^, ^54^ ^)^ The same group later reported that circulating levels of *miR*‐*19a*‐*3p* increased with age in older adults and decreased in centenarians compared to younger subjects.^(^ ^55^ ^)^ MicroRNAs are relatively stable in bodily fluids, including plasma, where they can be found enclosed in extracellular vesicles, or in a vesicle‐free state associated with proteins such as Argonaut 2 or high‐density lipoproteins.^(^ ^56^ ^)^ Although the remarkable stability of miRNAs in bodily fluids makes them valuable biomarkers for disease diagnosis and prognosis, it is debatable whether circulating miRNA levels can be used as a substitute for assessing their tissue level activity unless they can be traced back to their tissue of origin, which may explain the discordant findings.

Accumulation of senescent cells is known to contribute to aging and age‐related diseases including osteoporosis.^(^ ^16^, ^57^ ^)^ Although there are limited studies investigating the functional contributions of *miR*‐*19a*‐*3p* in cellular senescence, its role in attenuating age‐related bone loss has been well studied. Upregulation of *miR*‐*19a*‐*3p* is reported to reduce age‐induced bone loss in mice and increase osteogenesis in mouse BMSCs.^(^ ^35^ ^)^ Moreover, overexpression of *miR*‐*19a*‐*3p* is known to alleviate the progression of osteoporosis by upregulating *RUNX2* and *OCN* gene expression and enhancing alkaline phosphatase activity in human BMSCs.^(^ ^36^ ^)^ Nevola et al.^(^ ^58^ ^)^ noted that *miR*‐*19a*‐*3p* expression was higher in individuals without incident fracture compared to those with a fracture and was positively associated with increased bone mineral density and beta‐blocker usage. In addition to its role in attenuating age‐associated bone loss, we show that overexpression of *miR*‐*19a*‐*3p* also modestly attenuates the upregulation of key senescence effectors (i.e., *p16* ^*Ink4a*^ and *p21* ^*Cip1*^), enhances *Ki67* gene expression, which indicates increased proliferation, and decreases the number of SA‐β‐Gal+ cells, thus establishing its potential senotherapeutic role in bone by reducing senescence marker gene expression and decreasing cell proliferative arrest during physiological aging. These findings suggest that the decrease in *miR*‐*19a*‐*3p* observed in aging is consistent with its decrease in senescent cell culture models in vitro, where in both situations increased senescence is observed. Moreover, these data, combined with the previously established osteoanabolic role of *miR*‐*19a*‐*3p*, collectively suggest the clinical applicability of *miR*‐*19a*‐*3p* in attenuating the effects of bone aging and its associated skeletal diseases by inhibiting cellular senescence.

Our data are supported by a previous study in cardiomyocytes where increased expression of *miR*‐*19a*‐*3p* resulted in a reduced number of SA‐β‐Gal+ cells and downregulation of *p53* and *p21* expression in response to the induction of senescence using glucose.^(^ ^59^ ^)^ In addition to aging and senescence, *miR*‐*19a*‐*3p* overexpression has been known to inhibit the TLR3‐mediated nuclear factor kappa B activation and promote wound closure in mice by suppressing the release of inflammatory chemokines and cytokines by keratinocytes.^(^ ^60^ ^)^ This miRNA has also been affiliated with various types of cancer, such as those of bladder, gastric, pancreatic, lung, and colon (among others), thereby indicating its association with tumorigenesis.^(^ ^61^, ^62^, ^63^, ^64^, ^65^ ^)^ However, considering the interdependence between tumorigenesis and senescence,^(^ ^66^, ^67^ ^)^ it appears that this miRNA lies at the nexus of certain critical pathways that regulate both senescence and tumor growth and contributes to their transcriptional regulation.

Although we show that *miR*‐*19a*‐*3p* regulates *p21* ^*Cip1*^ levels in osteoblastic cells, *miR*‐*19a*‐*3p* is known to regulate a few other genes with known effects in bone. For example, *miR*‐*19a*‐*3p* promotes human BMSC differentiation through the suppression of *HDAC4*.^(^ ^36^ ^)^ *miR*‐*19a*‐*3p* has also been associated with beta‐blocker usage in humans and directly targets *ADRB1*.^(^ ^58^ ^)^ How regulation of these pathways influences cellular senescence through coregulation of *p21* ^*Cip1*^ will be the subject of future investigation.

A limitation of this study is the role of *miR*‐*19a*‐*3p* regulation of the p53 pathway, which was upregulated in our study, in contrast of its downregulation by *miR*‐*19a*‐*3p* expression in cardiomyocytes.^(^ ^59^ ^)^ While this could be simply due to differences in cell type (cardiomyocytes versus osteoblastic cells), it also could be a compensatory effect since the p53 pathway is a known upstream positive regulator of *p21* ^*Cip1*^, which was strongly downregulated by *miR*‐*19a*‐*3p*.

A major strength of this report is the discovery of the potential senotherapeutic role of *miR*‐*19a*‐*3p* in bone, by demonstrating its ability to suppress both *p16* ^*Ink4a*^ and *p21* ^*Cip1*^ gene expression and reduce SA‐β‐Gal activity following induction of senescence. It needs to be tested whether our results hold true in an in vivo setting where the miRNA does not act alone but is influenced by various other factors present in the bone microenvironment. Considering the multifaceted nature of *miR*‐*19a*‐*3p*, several hurdles/questions need to be addressed before its efficacy and safety can be established for clinical use: Does *miR*‐*19a*‐*3p* inhibit senescence in vivo and ultimately increase susceptibility to tumors? How does the physiological or disease state of the body modulate its role during aging or tumorigenesis? What are the various upstream and downstream factors that influence its function? Answers to these questions will be important in defining the role of *miR*‐*19a*‐*3p* in the regulation of senescence pathways during aging.

## Author Contributions

**Japneet Kaur:** Conceptualization; data curation; formal analysis; investigation; methodology; writing – original draft; writing – review and editing. **Dominik Saul:** Formal analysis; investigation; methodology; writing – review and editing. **Madison L. Doolittle:** Formal analysis; investigation; methodology; writing – review and editing. **Joshua N. Farr:** Methodology; supervision; writing – review and editing. **Sundeep Khosla:** Conceptualization; funding acquisition; project administration; supervision; writing – review and editing. **David G. Monroe:** Conceptualization; data curation; funding acquisition; investigation; methodology; project administration; supervision; writing – original draft; writing – review and editing.

## Conflict of Interest

The authors have nothing to disclose and no conflicts of interest.

### Peer Review

The peer review history for this article is available at https://www.webofscience.com/api/gateway/wos/peer‐review/10.1002/jbm4.10745.

## Supporting information


**Fig. S1.** RT‐qPCR analysis of miR‐106b‐5p following induction of senescence using (*A*) etoposide, (*B*) H_2_O_2_, and (*C*) passaging. Values of *p* are shown numerically with *p* < 0.05 (independent samples *t* test).
**Fig. S2.** Representative images of SA‐β‐Gal‐stained CalOBs treated with (*A*) vehicle (DMSO) and etoposide (20 uM) and (*B*) control (untreated) and H_2_O_2_ (magnification ×10; *n* = 3/group). RT‐qPCR analysis of (*C*, *D*) *p16* ^*Ink4a*^ and *p21*_*Cip1*_ in nonsenescent and senescent CalOBs. Gene expression was denoted as fold‐change relative to vehicle or control (*n* = 3/group). Values of *p* are shown numerically with *p* < 0.05 (independent samples *t* test).
**Fig. S3.** (*A*, *B*) Heatmaps corresponding to enriched gene sets identified using GSEA showing differential expression (using normalized counts) of genes included in those gene sets (only genes with *p*_adjus_ < 0.05 and log_2_ fold‐change > [1] are shown for visualization purposes). (*C*) Table shows log_2_ fold‐change values (miR‐19a‐3p mimic/control) and *p* values for genes shown in Fig. 3*G*.

**Table S1.** GSEA of RNAseq data from CalOBs transfected with either negative control or miR‐19a‐3p miRNA mimics. NES = normalized enrichment score, *p*.adjust = adjusted *p* values.

**Table S2.** KEGG analysis of RNAseq data from CalOBs transfected with either negative control or miR‐19a‐3p miRNA mimics. NES = normalized enrichment score, *p*.adjust = adjusted *p* values.

**Table S3.** TargetScanMouse 8.0 predicted miR‐19a‐3p target genes, with associated gene expression data from RNAseq (CalOBs transfected with either negative control or miR‐19a‐3p miRNA mimics). NES = normalized enrichment score, *p*.adjust = adjusted *p* values.

## Data Availability

The data that support the findings of this study are available from the corresponding author upon reasonable request.
