# Supplementary material for: MicroRNA‐19a‐3p Decreases with Age in Mice and Humans and Inhibits Osteoblast Senescence
Source: JBMR Plus. 2023 Apr 18;7(6):e10745. doi: 10.1002/jbm4.10745 (PMC10241091; doi:10.1002/jbm4.10745)
Supplement: Supplementary file 1 — Fig. S1. RT‐qPCR analysis of miR‐106b‐5p following induction of senescence using (A) etoposide, (B) H2O2, and (C) passaging. Values of p are shown numerically with p < 0.05 (independent samples t test). Fig. S2. Representative images of SA‐β‐Gal‐stained CalOBs treated with (A) vehicle (DMSO) and etoposide (20 uM) and (B) control (untreated) and H2O2 (magnification ×10; n = 3/group). RT‐qPCR analysis of (C, D) p16 Ink4a and p21 Cip1 in nonsenescent and senescent CalOBs. Gene expression was denoted as fold‐change relative to vehicle or control (n = 3/group). Values of p are shown numerically with p < 0.05 (independent samples t test). Fig. S3. (A, B) Heatmaps corresponding to enriched gene sets identified using GSEA showing differential expression (using normalized counts) of genes included in those gene sets (only genes with p adjus < 0.05 and log2 fold‐change > [1] are shown for visualization purposes). (C) Table shows log2 fold‐change values (miR‐19a‐3p mimic/control) and p values for genes shown in Fig. 3G . [file JBM4-7-e10745-s001.pdf]

Supplementary Material

MicroRNA-19a-3p decreases with age in mice and humans and inhibits osteoblast senescence

Authors: Japneet Kaur[1,2], Dominik Saul[1,2], Madison L. Doolittle[1,2], Joshua N. Farr[1,2], Sundeep Khosla[1,2], and David G. Monroe[1,2*]

Supplementary Material Figure Legends

Suppl. Figure 1. RT–qPCR analysis of *miR-106b-5p* following induction of senescence using **(A)** etoposide, **(B)** H_2O_2, and **(C)** passaging. Values of p are shown numerically with $p < 0.05$ (independent samples *t*-Test).

Suppl. Figure 2. Representative images of the SA-β-Gal stained CalOBs treated with **(A)** vehicle (DMSO) and etoposide (20uM), and **(B)** control (untreated) and H_2O_2 (magnification, 10X; n = 3/group). RT–qPCR analysis of **(C, D)** $p16^{Ink4a}$ and $p21^{Cip1}$ in non-senescent and senescent CalOBs. Gene expression was denoted as fold-change relative to vehicle or control (n = 3/group). Values of p are shown numerically with $p < 0.05$ (independent samples *t*-Test).

Suppl Figure 3. (A, B) Heatmaps corresponding to the enriched gene sets identified using GSEA showing differential expression (using normalized counts) of the genes included in those gene sets (only genes with Padjus < 0.05 and $\log_2$ fold-change > [1]

are shown for visualization purposes). **(C)** Table shows the log_2 fold-change values (*miR-19a-3p mimic /* Control) and p-values for genes shown in Figure 3G.

Suppl. Figure 1

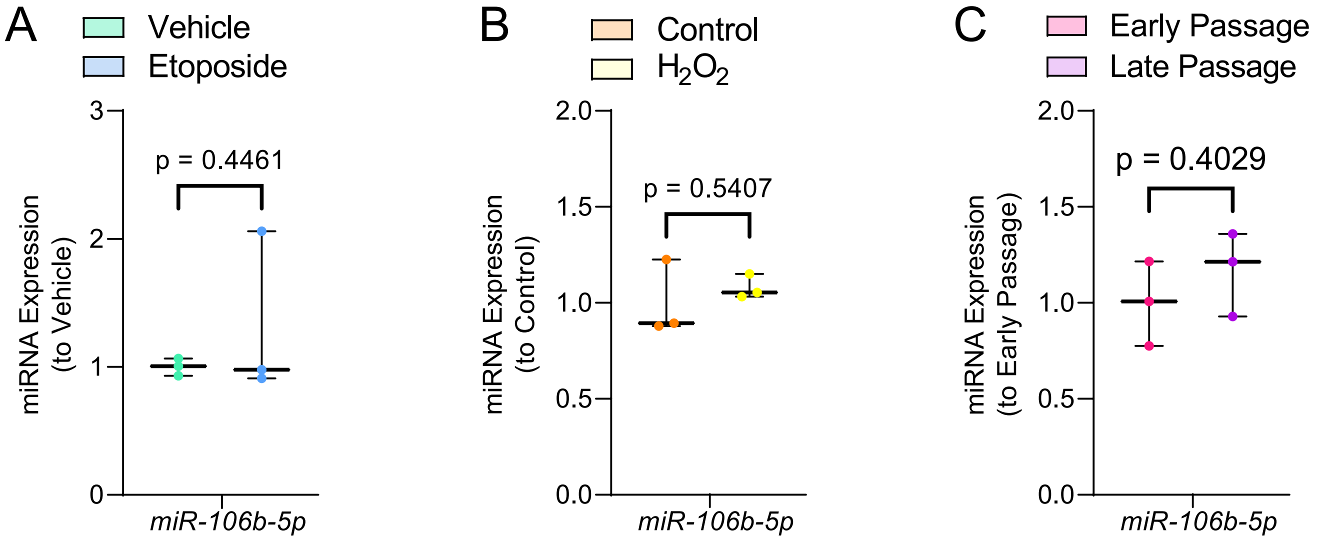

Suppl. Figure 2

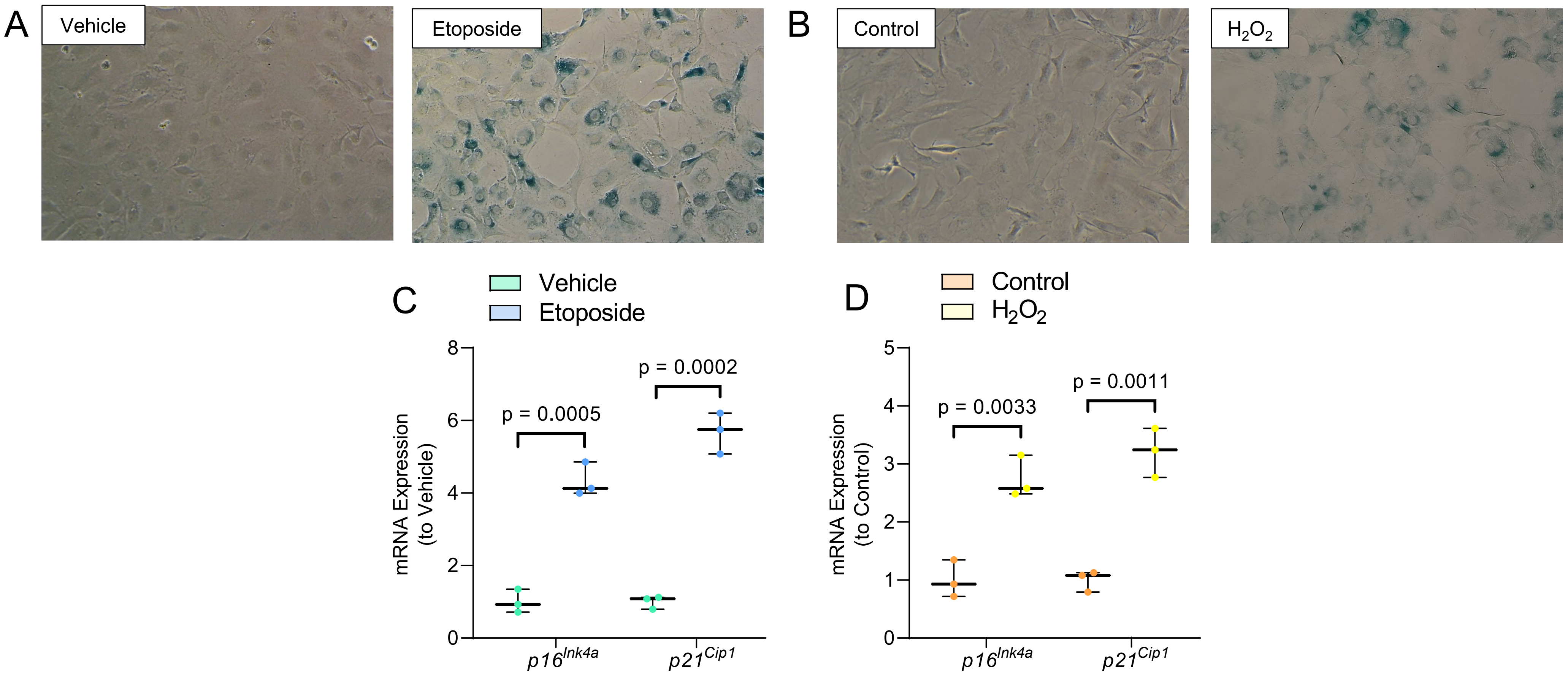

Suppl Figure 3

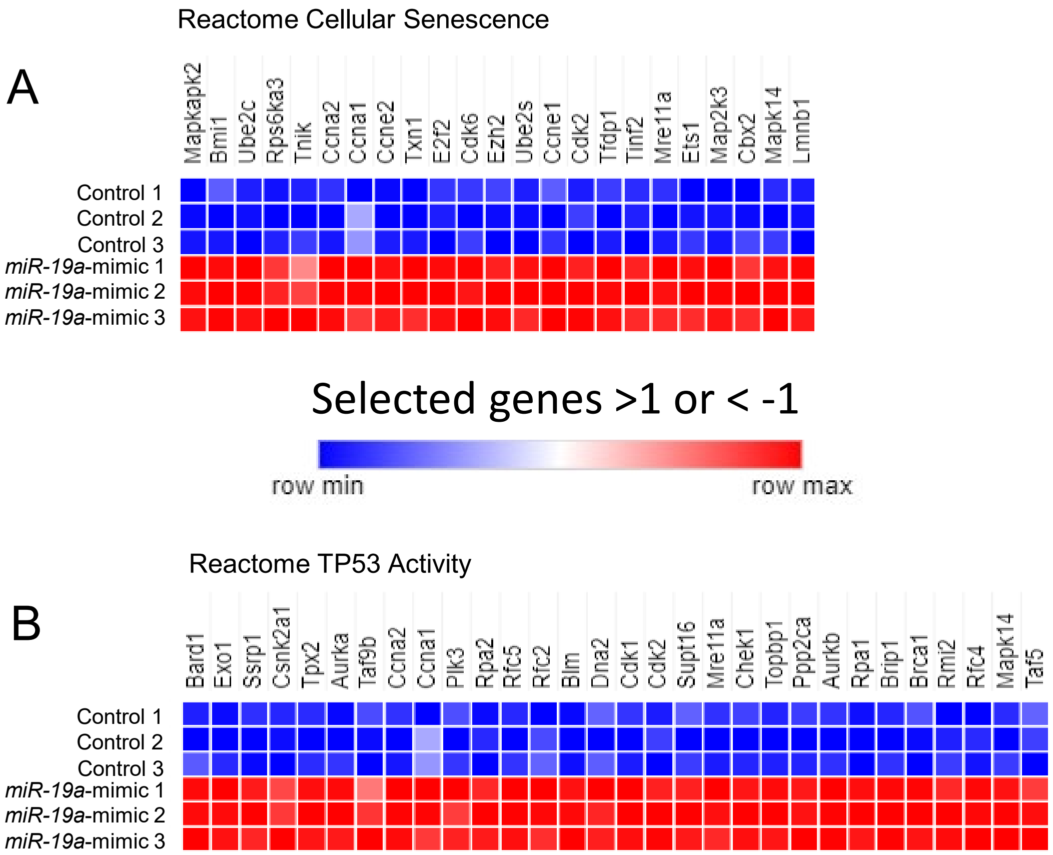

C

Gene	Log_2 Fold Change	P-value	P_{adjus}
Cdkn1a	-1.451	4.3E-170	3.3E-168
Jak3	-1.051	1.9E-24	1.5E-23
Cdkn2b	-0.840	6.2E-29	5.8E-28
Stat1	-0.667	4.4E-13	2.3E-12
Stat2	-0.439	2.1E-13	1.1E-12
Stat3	-0.379	7.0E-20	4.8E-19
Tyk2	-0.366	2.6E-08	1.0E-07
Ccnd1	0.261	2.2E-08	8.7E-08
Jak1	0.291	1.8E-12	8.7E-12
Cdk4	0.355	1.1E-14	5.9E-14
Jak2	0.416	5.9E-10	2.5E-09
E2f1	0.505	5.1E-16	3.0E-15
E2f5	0.555	1.6E-11	7.7E-11
E2f4	0.646	2.6E-33	2.7E-32
E2f6	0.777	4.0E-48	6.1E-47
E2f3	0.875	1.8E-42	2.4E-41
Ccnd2	1.086	3.5E-183	3.1E-181
Ccnd3	1.107	2.1E-70	5.0E-69
Cdk2	1.125	1.4E-73	3.5E-72
Bmi1	1.175	2.0E-76	5.2E-75
Cdk6	1.689	1.4E-131	7.3E-130
Ccne1	2.213	5.1E-115	2.2E-113
Ccne2	2.892	2.1E-196	2.1E-194
E2f2	3.478	2.7E-97	9.4E-96
E2f8	3.527	4.0E-150	2.5E-148
E2f7	3.650	6.7E-65	1.4E-63
Lmnb1	2.746	0.000	0.000
